# Evaluation of the Impact of the Sustainable Development Goals on an Activity Recognition Platform for Healthcare Systems

**DOI:** 10.3390/s23073563

**Published:** 2023-03-29

**Authors:** José L. López, Macarena Espinilla, Ángeles Verdejo

**Affiliations:** 1 Computer Science Department, University of Jaén, Campus Las Lagunillas s/n, 23071 Jaén, Spain; 2 Electrical Engineering Department, University of Jaén, Campus Las Lagunillas s/n, 23071 Jaén, Spain; mverdejo@ujaen.es

**Keywords:** sustainable development goals (SDGs), healthcare platform, activity recognition, sensor devices

## Abstract

The Sustainable Development Goals (SDGs), also known as the Global Goals, were adopted by the United Nations in 2015 as a universal call to end poverty, protect the planet and ensure peace and prosperity for all by 2030. The 17 SDGs have been designed to end poverty, hunger, AIDS and discrimination against women and girls. Despite the clear SDG framework, there is a significant gap in the literature to establish the alignment of systems, projects or tools with the SDGs. In this research work, we assess the SDG alignment of an activity recognition platform for healthcare systems, called ACTIVA. This new platform, designed to be deployed in environments inhabited by vulnerable people, is based on sensors and artificial intelligence, and includes a mobile application to report anomalous situations and ensure a rapid response from healthcare personnel. In this work, the ACTIVA platform and its compliance with each of the SDGs is assessed, providing a detailed evaluation of SDG 7—ensuring access to affordable, reliable, sustainable and modern energy for all. In addition, a website is presented where the ACTIVA platform’s compliance with the 17 SDGs has been evaluated in detail. The comprehensive assessment of this novel platform’s compliance with the SDGs provides a roadmap for the evaluation of future and past systems in relation to sustainability.

## 1. Introduction

The World Health Organization (WHO) has published a report on the occasion of the United Nations declaring the Decade of Healthy Aging 2020–2030. This document provides an overview of the world’s population and its needs, particularly in terms of the quality of life [1]. The beginning of the 2020s coincided with the COVID-19 pandemic, which has significantly affected the elderly.

Healthy aging is a process of developing and maintaining functional capacity and enabling well-being in old age. Therefore, the goal of the decade is to optimize the functional capacity of the elderly, where technology, software, hardware, engineering and their intelligent applications will play an important role [2,3,4]. An essential aspect in smart environments is data management and the integration of sensor installations, Internet of Things and equipment without disrupting people’s daily lives and without creating a very marked digital divide for the elderly, disabled or vulnerable. Cyber, physical and social systems play a key role in people’s daily activities [5].

Human activity recognition (HAR) systems monitor activity patterns and intervene when a change in behavior or a critical event has occurred [6,7,8,9,10]. These systems are based on complex architectures that collect data with sensor devices, performing computations and extracting knowledge in order to obtain relevant activity information [11,12,13]. Thus, HAR systems are becoming crucially valuable, as they are able to help elderly or vulnerable people to live more independently [14].

Multiple proposals [15,16] indicate that industry and social institutions and systems must offer multi-sectoral clusters to rise to the challenge of achieving healthy aging, together with sustainability and energy efficiency, through the application of intelligent systems [16,17]. There have been multiple recent studies that highlight the gap in analyzing and implementing the Sustainable Development Goals (SDGs) in the fields of engineering, computer science, technology and healthcare [18,19,20,21].

This paper addresses the need for an in-depth and practical analysis of the SDGs in relation to HAR systems in the ACTIVA platform [22], where the fields of engineering, computer science, technology and health converge to propose a solution to the elderly [23,24]. The ACTIVA platform [22] is presented in this paper as a smart platform that deploys non-invasive sensors in environments inhabited by elderly or vulnerable people. It includes a mobile application to send notifications to caregivers or family members to enable real-time activity recognition in a multi-occupant environment, while preserving user privacy. This platform has been registered at the national level and successfully deployed in a real-world setting, specifically in a nursing home during the pandemic.

The main objective of this paper is to evaluate how the ACTIVA platform aligns with the SDG targets and indicators, quantitatively and qualitatively, by means of the taxonomy proposed in [21]. Specifically, this paper includes a global assessment of all sustainable development goals and a specific assessment of SDG 7–ensuring access to affordable, reliable, sustainable and modern energy for all. In addition, a website is presented, where all SDGs are assessed. Therefore, the main contributions of our study are the following:

- Our research presents the ACTIVA platform, which is an intelligent activity recognition system designed to monitor aging populations. Its main objective is to improve the quality of life of elderly people who have a certain degree of physical or cognitive dependency. The system can be extrapolated to multiple environments or sectors such as assisted living environments for young people with cognitive limitations.
- A quantitative analysis of the ACTIVA platform is performed based on the SDGs. We present a global assessment of all sustainable development goals and a detailed specific assessment of SDG 7. Furthermore, a website is presented, where all SDGs are assessed with the same level of detail as SDG 7, including an overall evaluation for each objective. Finally, a roadmap for increasing compliance with the SDGs in the future is proposed.

The assessment of this platform against the SDGs will enhance its prestige from a business, institutional and social point of view. The quantification also allows us to continuously monitor and improve the systems in place based on a vision of the future [25,26,27].

The rest of the paper is organized as follows: Section 2 reviews the sustainable development goals and the existing tools for their evaluation. Section 3 describes the ACTIVA platform based on human activity recognition in environments inhabited by elderly. Section 4 explains the quantitative and qualitative analysis of the ACTIVA platform based on the SDGs. Section 5 outlines the main conclusions obtained.

## 2. Review of the Sustainable Development Goals

In 2015, the member states of the United Nations agreed to establish an agenda of actions aimed at reducing pollution, improving the health and quality of life of the world’s population and enhancing environmental sustainability [28].

The agenda includes 17 SDGs, the fulfillment of which requires us to evaluate any industrial, human, biological, social, economic or other type of action that affects our planet. Thus, studies identify six key pillars for the SDGs [29]:

1. Education, gender and inequality.
2. Health, wellness and demographics.
3. Energy decarbonization/sustainable industry.
4. Sustainable food, land, water and oceans.
5. Sustainable cities and communities.
6. Digital revolution for sustainable development.

In the literature, tools have been presented to assess the 17 SDGs. The most relevant are described in the Table 1.

**Figure 1:**
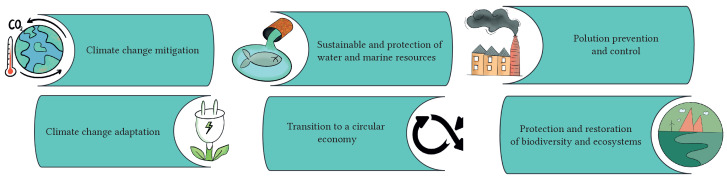
European Union taxonomy areas to be assessed in SDG.

Some of the taxonomies and principles addressing SDG alignment in the field of technology and its impact on society, such as the report on the European Union Taxonomy for Sustainable Activities, will be taken as essential references [38]. This source provides an update to the technical selection criteria for 70 climate change mitigation activities and 68 adaptation activities, as shown in Figure 1.

The application of the taxonomy to industrial activities or any other type of development, design or system within the EU will lead to a direct improvement in the financing and achievement of business objectives, which will be validated in the future development of the project and its implications for society.

**Figure 2:**
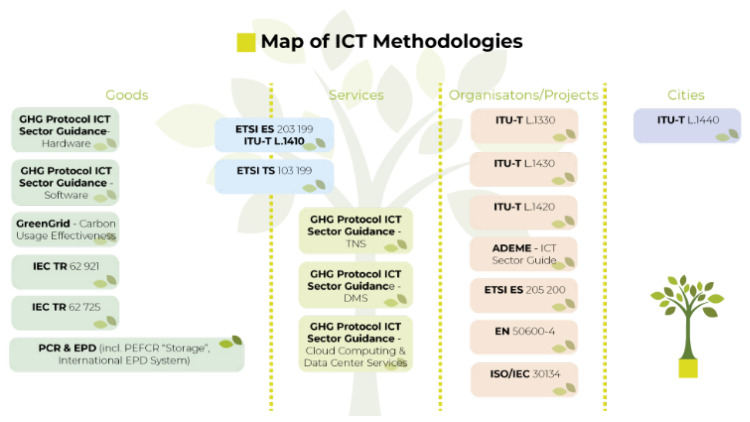
Assessment blocks in the European Union taxonomy.

Another reference for this research work is the analysis of intelligent systems reviewed in the reports of the European Framework Initiative for Energy and Environmental Efficiency in the ICT Sector [40] (Figure 2). We have also drawn on research such as Pereira’s, which establishes an analysis of energy efficiency and software languages [41].

Most of the research analyzed on the SDG alignment with systems, buildings or environments focuses on the analysis of one or several goals. However, in this work we have taken a broader approach through a heterogeneous sustainability assessment procedure based on the 2030 Agenda, in which all the SDGs and 231 indicators have been evaluated [28,35].

As detailed in [42], smart cities and living laboratories used for experimentation, such as our system, are spaces that go beyond the concept of buildings or indoor environments.

In this paper, the taxonomy proposed in [21] has been selected to assess the ACTIVA platform’s alignment due to its similarity with the ACTIVA platform.

## 3. ACTIVA Platform

In this section, we provide a detailed overview of the ACTIVA platform for activity recognition in the context of healthcare systems. To do so, first, Section 3.1 presents the scenario where the platform has been deployed (a nursing home). Section 3.2 presents the sensor devices used in the ACTIVA platform and data acquisition. The methodology used to detect the subject’s location and daily activities is presented in Section 3.3, along with the mobile app for caregivers in the elderly care home.

### 3.1. Domain: Elderly Care Home

The ACTIVA platform has been designed, implemented and tested in an elderly care home in Alcaudete (a town in the Spanish province of Jaén). In our scenario, seven elderly people were monitored through the system with the involvement of two caregivers who used ACTIVA to monitor their activities and locations.

Specifically, four double rooms on the same floor of the care home were monitored. Every inhabitant has a shared room with another resident. This space consists of a bathroom, a small entrance and a bedroom. In the bedroom, each resident has their own individual space: closet, bedside table, bed and other items such as chairs and shelves. This area is 6.67 m wide and 3.04 m high, with a total floor area of 20.28 m${}^{2}$. Moreover, the inhabitant has access to common areas shared by all the elderly residents outside the room, where they perform other activities, such as eating, physical exercise and cognitive exercises.

The aim of the ACTIVA platform is to detect the location and daily activities of the elderly person within a shared room, which is the place with the greatest risk of the person suffering any anomaly.

Considering each inhabitant’s personal area, and taking into account the location of the inhabitant within this zone, the platform is able to distinguish between a series of general locations: bathroom, bedroom and outside the room. As for daily activity recognition, the target activities are the following:

- In the bathroom: use the toilet and take a shower;
- In the bedroom: dress and sleep;
- Exit bedroom.

### 3.2. Sensor Devices and Data Acquisition

In order to collect the location and activity of the subject, it is necessary to deploy a series of smart devices equipped with sensors [43,44].

Although various sensor devices have been presented in the literature for such purposes, the ACTIVA platform has been deployed with a device-free approach. This ensures data input without the need for the elderly person to interact directly with the devices—a desirable feature considering that the subjects to be monitored are elderly people. In addition, the small size of the technology entails minimum changes to their environment, which makes it practically invisible and non-invasive.

**Figure 3:**
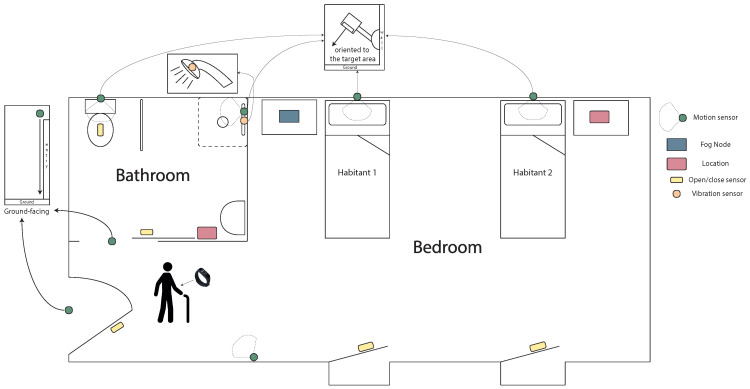
Location of the devices in each shared room.

The selected devices have been proven to be successful in recent activity recognition methodologies [4,11,45]. They can be divided into two groups according to their purpose: location or daily activity detection. Figure 3 illustrates the location of the devices in each shared room.

On the one hand, location detection is based on RSSI values using BLE. The RSSI value usually oscillates between [0,−100], and indicates approximately whether a device with BLE connection is closer or farther away from the other BLE device that is scanning it. The higher the value (closer to 0), the better the BLE connection, and therefore the closer it is. If the figure is lower (closer to −100), the two devices are farther away. By combining several BLE devices in a given space, we can locate a device within it. The ACTIVA platform uses the following location devices:

- Activity wristband. This wristband is a Xiaomi Mi Band 3 that is equipped with Bluetooth Low Energy 4.2 (BLE v4.2), and from which we obtain a Received Signal Strength Indicator (RSSI) at all times. Although this device is used for location, its battery level and the steps taken by the inhabitant are also obtained.
- Location anchor [46]. Small computing unit that also has BLE connection. Its purpose is to scan its assigned target beacons to obtain their RSSI value.

On the other hand, the sensor devices [47] for activity recognition are the following:

- Motion sensor. Binary device that indicates whether or not movement is occurring within its range.
- Open/close sensor. Binary device usually placed on doors or drawers to indicate closed or open status.
- Vibration sensor. A device (located on an object) which indicates the occurrence of a vibration caused by movement of the object with which it is associated.

Finally, a local node (another small computing unit) collects all the RSSI values sampled by each of the location anchors by means of the MQTT protocol, and the sensor data by Zigbee. The data acquired in the local node are sent to the server to apply the activity recognition methodology that are proposed in the following section.

### 3.3. HAR Methodology and Mobile App

The HAR methodology in the ACTIVA platform for a target inhabitant follows the approach below:

1. New samples sent by the local node are received. RSSI, sensor updates, and step counts are introduced in a temporary cache for the last minute samples, and data that have exceeded that time limit are deleted.
2. Location recognition. A time window of 10 s is applied with the maximum operator to the received RSSI data, based on a proposal found in the literature [8,13,48]. The value resulting from applying the aggregation function is used as input data for our previously trained machine learning model, thus generating the location.
3. Activity recognition. After the location has been inferred, a set of fuzzy rules [49] that take into account the sensor data is applied to determine the activity. This inference must be checked against the location. If the location with which the activity is associated does not match, it is determined that the inhabitant is not performing the activity.

The location and activity are persistently stored in the server, which is connected to the ACTIVA mobile app used by caregivers to monitor the inhabitants. This app is essential in current times, where everyone has a mobile phone that allows intercommunication with a full set of equipment and devices [7,12,50].

**Figure 4:**
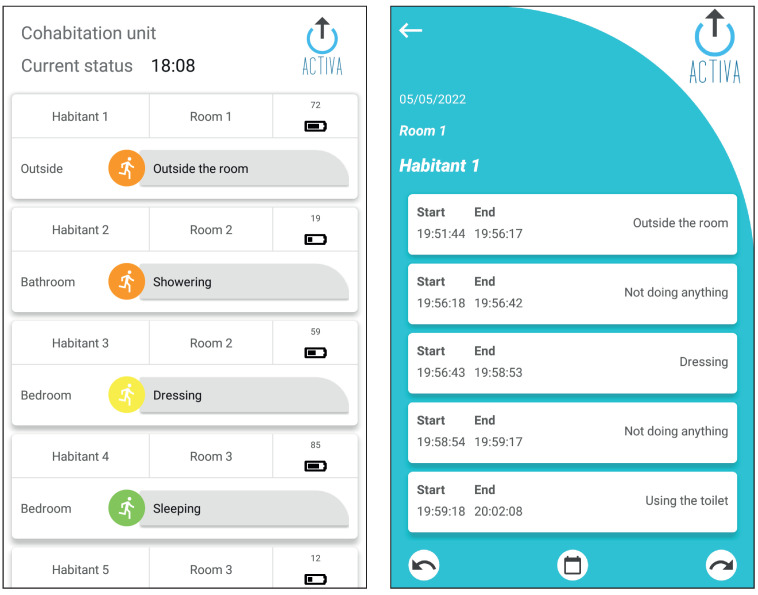
ACTIVA mobile application. The left image shows the inhabitants list view and the right image shows the list of historical activities of an inhabitant.

The ACTIVA mobile app offers caregivers the following functionalities (Figure 4):

- Inhabitant list view. A list of inhabitants is displayed when starting the application (illustrated in Figure 4). For each inhabitant, the last detected location and activity, their room, and the battery level of their activity wristband are displayed. All this information is updated in real time. The name of the elderly person and their room are displayed anonymously, so that only the caregivers know who each inhabitant is.
- Inhabitant activities view. A list of activities detected for each inhabitant is displayed in chronological order (illustrated in Figure 4). It only shows the activities on a specific day (by default, the current date of the mobile device). The caregiver can access the activities from other days through the calendar, or go to previous or subsequent days by using the arrows.
- Notifications. The caregiver is notified of any changes in the inhabitant’s activity, using different colors to distinguish between activities according to the associated danger level. It should be noted that notifications are customizable from the dashboard.
  - High danger. The risk of unexpected situations, such as falling in the shower, is high.
  - Moderate danger. The risk is moderate or very improbable; for example, the person has been up at night and has not gone back to bed for more than 10 min.
  - Low danger. The risk is very low or there is no danger at all.

## 4. Analysis of the ACTIVA Platform with SDGs

Once the research environment and its main operational and equipment characteristics were determined in the ACTIVA platform, the evaluation is presented in the application of a taxonomy that has already been successfully implemented in the previous work [21].

First, we present the detailed evaluation of the ACTIVA platform’s compliance with SDG 7 in Section 4.1. Then, we present the website, where compliance with the rest of the SDGs is detailed in Section 4.2, and, finally, we present a general discussion that takes into account the evaluation of each of the goals in the ACTIVA platform, in Section 4.3.

### 4.1. Detailed Assessment of SDG 7

Table 2 shows a detailed analysis the ACTIVA platform’s compliance with SDG 7: Ensure access to affordable, reliable, sustainable and modern energy for all. The first column shows the targets and the second column shows the indicators, according to the parameters taken from the United Nations report, Global Indicator Framework for Sustainable Development Goals [35]. Furthermore, Table 2 is structured to quantify the SDG indicators and their alignment with the ACTIVA platform, as well as the main systems installed and lines of action.

### 4.2. Website for Assessment of all SDGs

**Figure 5:**
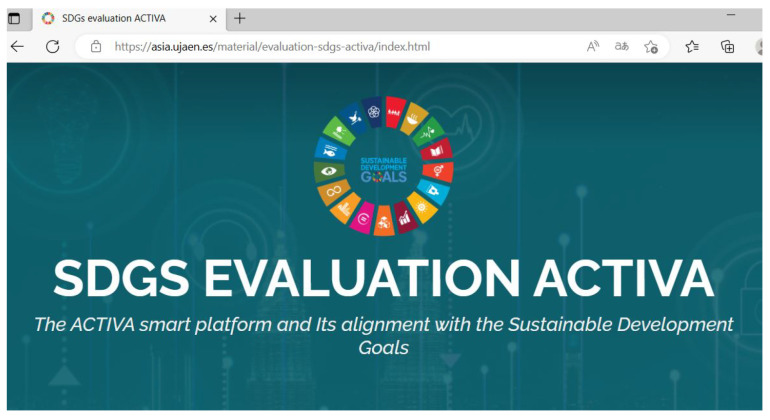
Website SDGs and ACTIVA platform.

In this section, we present the website (see Supplementary Materials) that collects all the evaluated SDGs, as illustrated in Figure 5.

**Figure 6:**
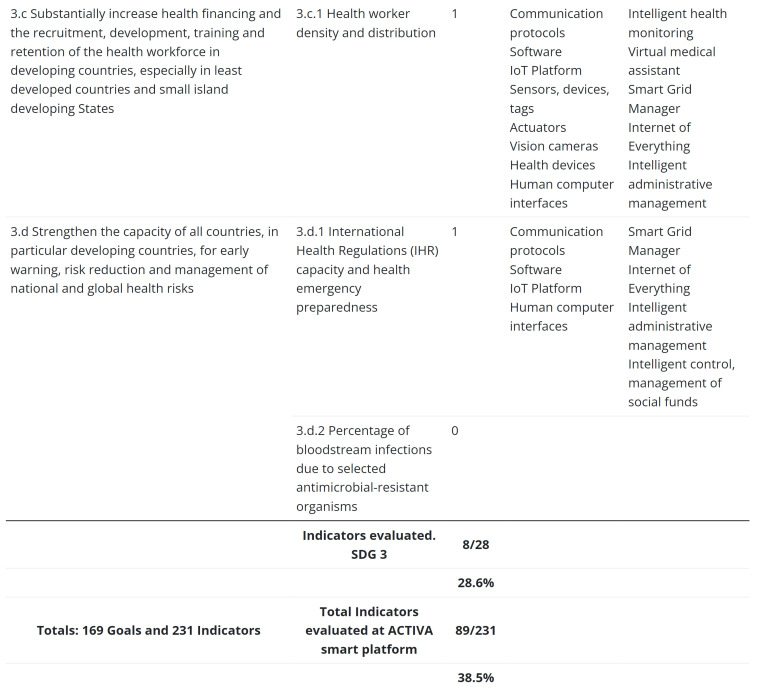
An excerpt from the SDG 3 assessment on the website.

The SDG targets and indicators are assessed in detail in the website with the aim of developing an implementation model for smart platforms and systems. The ACTIVA platform has reached 28.6% alignment with SDG 3, which refers to health and well-being. Figure 6 shows an excerpt from the assessment of SDG 3 on the website. In this case, we have to point out that some of the objectives of this SDG target the institutional level, which is out of our system’s scope.

### 4.3. Global Analysis and Roadmap

The main objective is to quantify and examine how the ACTIVA platform performs in terms of sustainability and present a roadmap for increasing compliance with the SDGs in the future.

A summary of the results, which are described in detail in the website, is shown in Table 3. We can observe, for example, that nine of the indicators are fulfilled in SDG 11, achieving 60% alignment. Conversely, with SDG 14, related to the protection of the oceans, the ACTIVA platform does not comply with any of the indicators, so its alignment percentage is 0%.

With this detailed evaluation of an innovative platform, this paper proposes a clear example of how to perform an SDG compliance evaluation with the aim of enhancing the innovation and development of smart systems, sensors, and devices that can contribute to the environmental, social, economic, and health sustainability of people and the environment.

Finally, continuous improvement in sustainability is one of the parameters to be taken into account in the ACTIVA platform. Therefore, in addition to submitting the ACTIVA platform to the alignment of the SDGs, we also propose a roadmap for the platform to evolve and meet indicators in the coming years through future planning and strategy.

**Figure 7:**
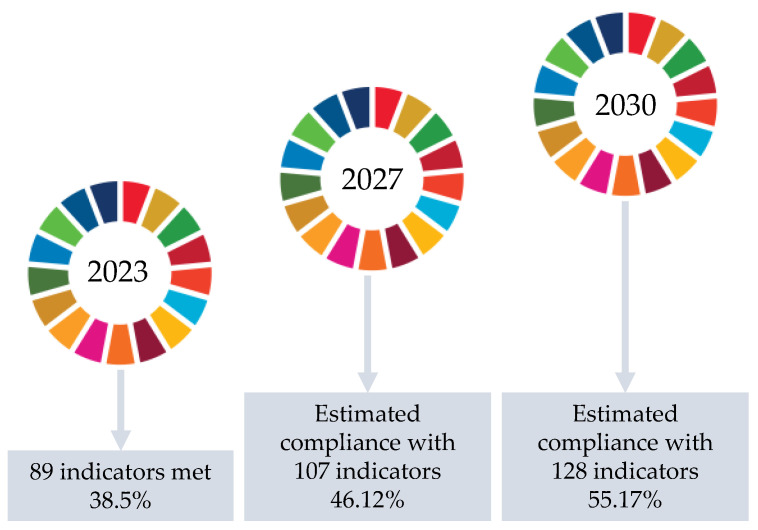
Sustainability roadmap on the ACTIVA platform.

The ACTIVA platform roadmap prioritizes the inclusion of new developments that are efficient and sustainable. Thus, the goal is to exceed the percentage of compliance with the SDG indicators by more than 50% (Figure 7). To this end, we must prioritize and reinforce the objectives that are key to the ACTIVA platform. First, we strive to improve SDG 3 by 20% in 2027 and 40% by 2030, expanding the health devices integrated in ACTIVA and updating the existing ones. Secondly, we strive to improve SDG 8 by adding technologies to support the work of specialized personnel. Thirdly, we strive to strengthen SDG 9 by fostering innovation in agencies and institutions using the ACTIVA platform. Finally, SDG 11 can be improved by implementing the platform in rural or disadvantaged territories, among others.

## 5. Conclusions

There are multiple recent studies that highlight the gap in analyzing and implementing the Sustainable Development Goals in the fields of engineering, computer science, technology, and healthcare. To reduce this gap, this paper has presented ACTIVA, an activity recognition platform deployed in a nursing home where elderly people are being monitored by healthcare professionals through a mobile app.

A quantitative analysis has been performed to assess the ACTIVA platform’s compliance with the SDGs. To provide an example of this assessment, a detailed evaluation of SDG 7 for the ACTIVA platform has been presented. Then, the website that details the evaluation of all the SDGs has been presented and, finally, a global evaluation has been carried out that takes into account all the SDGs assessed. With a detailed evaluation of an innovative platform, this paper proposes a clear example of how to evaluate future and past systems according to the standards proposed by the SDGs and their indicators, with the aim of improving innovation and development.

## Figures and Tables

**Table 1:** SDG Assessment Tools.

Tool	Description	Reference
SDG Compass	Management strategy for the achievement of the SDGs	[30]
SDSN Northern Europe	United Nations Sustainable Development Solutions Network (SDSN) Northern Europe	[31]
IRIRS +	Analysis and evaluation tool using taxonomies for measuring environmental impacts	[32]
PNUD	United Nations Development Programme (UNDP)	[33]
KTH’s Toolbox	KTH’s Toolbox for Learning for Sustainable Development offers tips and examples for the integration of sustainable development into teaching	[34]
Local 2030	Local 2030: localizing the SDGs, is an online platform. Stakeholders and partners share tools, experiences, new solutions and guides to support SDG localization	[35]
SDG Toolkit	SDG Toolkit is part of a European project to involve European NGOs at national and European level in the SDGs.	[36]
SDG Helpdesk	SDG Helpdesk provides access to models, methodologies and guidance to support policy makers in the implementation of the SDGs	[37]
EU taxonomy	The EU taxonomy is a classification system which establishes a list of environmentally sustainable economic activities	[38]
SDG Smart Labs	Assessment of SDGs in Smart Labs	[39]

**Table 2:** Evaluation and implementation of SDG 7 in the ACTIVA smart platform.

Global Indicator Framework for the Sustainable Development Goals and Targets of the 2030 Agenda for Sustainable Development			Evaluation of SDGs in the ACTIVA SMART PLATFORM	
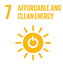	**Goal 7. Ensure Access to Affordable, Reliable, Sustainable, and Modern Energy for all**			
**Goals and Targets**	**Indicators**	**Existing**	**Installed Devices/Systems**	**Field/Area of Action**
7.1 By 2030, ensure universal access to affordable, reliable, and modern energy.	7.1.1 Proportion of population with access to electricity.	1	Communication protocols. Software. IoT Platform. Sensors, devices, and tags. Actuators. Human computer interfaces.	Social assistance. Smart Grid Manager. Energy control. Renewable energy. Internet of Energy. Economic and environmental sustainability. Intelligent administrative management. Intelligent control, management of social funds. Safety.
	7.1.2 Proportion of population with primary reliance on clean fuels and technology.	1		
7.2 By 2030, increase substantially the share of renewable energy in the global energy mix.	7.2.1 Renewable energy share in the total final energy consumption.	1		
7.3 By 2030, double the global rate of improvement in energy efficiency.	7.3.1 Energy intensity measured in terms of primary energy and GDP.	1		
7.a By 2030, enhance international cooperation to facilitate access to clean energy research and technology, including renewable energy, energy efficiency and advanced and cleaner fossil-fuel technology, and promote investment in energy infrastructure and clean energy technology.	7.a.1 International financial flows to developing countries in support of clean energy research and development and renewable energy production, including in hybrid systems.	1	Communication protocols. Software. IoT Platform. Human computer interfaces. Renewable energy. Internet of Energy. Economic.	Environmental sustainability. Intelligent administrative management. Intelligent control, management of social funds.
	Indicators evaluated. SDG 7	6/6		
		100%		

**Table 3:** Indicators evaluated in ACTIVA.

	SDG	Existing	%
1	End poverty in all its forms everywhere	5	38.5
2	End hunger, achieve food security, and improved nutrition and promote sustainable agriculture	3	21.4
3	Ensure healthy lives and promote well-being for all at all ages	8	28.6
4	Ensure inclusive and equitable quality education and promote lifelong learning opportunities for all	6	50.0
5	Achieve gender equality and empower all women and girls	8	57.1
6	Ensure availability and sustainable management of water and sanitation for all	6	54.5
7	Ensure access to affordable, reliable, sustainable, and modern energy for all	6	100.0
8	Promote sustained, inclusive, and sustainable economic growth, full and productive employment and decent work for all	3	18.8
9	Build resilient infrastructure, promote inclusive and sustainable industrialization and foster innovation	7	58.3
10	Reduce inequality within and among countries	2	14.3
11	Make cities and human settlements inclusive, safe, resilient, and sustainable	9	60.0
12	Ensure sustainable consumption and production patterns	2	15.4
13	Take urgent action to combat climate change and its impacts	3	37.5
14	Conserve and sustainably use the oceans, seas, and marine resources for sustainable development	0	0.0
15	Protect, restore and promote sustainable use of terrestrial ecosystems, sustainably manage forests, combat desertification, and halt and reverse land degradation and halt biodiversity loss	1	7.1
16	Promote peaceful and inclusive societies for sustainable development, provide access to justice for all, and build effective, accountable and inclusive institutions at all levels	7	29.2
17	Strengthen the means of implementation and revitalize the global partnership for sustainable development	13	54.2
	Total	89	38.5

## Data Availability

The data presented in this study are available in Supplementary Materials.

## Supplementary Materials

An additional table has been prepared, detailing the 17 SDGs together with their targets and indicators and their influence on the evaluated parameters of the ACTIVA smart platform. This material can be reviewed on the website: https://asia.ujaen.es/material/evaluation-sdgs-activa/index.html.
